# New Insights in the Interplay Between African Swine Fever Virus and Innate Immunity and Its Impact on Viral Pathogenicity

**DOI:** 10.3389/fmicb.2022.958307

**Published:** 2022-07-06

**Authors:** Abraham Ayanwale, Sascha Trapp, Rodrigo Guabiraba, Ignacio Caballero, Ferdinand Roesch

**Affiliations:** UMR 1282 ISP, INRAE Centre Val de Loire, Nouzilly, France

**Keywords:** ASFV, CGAS, NFkapapB, innate immunity, STING

## Abstract

The continuous spread of African swine fever virus (ASFV) in Europe and Asia represents a major threat to livestock health, with billions of dollars of income losses and major perturbations of the global pig industry. One striking feature of African swine fever (ASF) is the existence of different forms of the disease, ranging from acute with mortality rates approaching 100% to chronic, with mild clinical manifestations. These differences in pathogenicity have been linked to genomic alterations present in attenuated ASFV strains (and absent in virulent ones) and differences in the immune response of infected animals. In this mini-review, we summarized current knowledge on the connection between ASFV pathogenicity and the innate immune response induced in infected hosts, with a particular focus on the pathways involved in ASFV detection. Indeed, recent studies have highlighted the key role of the DNA sensor cGAS in ASFV sensing. We discussed what other pathways may be involved in ASFV sensing and inflammasome activation and summarized recent findings on the viral ASFV genes involved in the modulation of the interferon (IFN) and nuclear factor kappa B (NF-κB) pathways.

## Introduction

Since its identification in 1921 in Kenya (Eustace Montgomery, 1921), African swine fever (ASF) has been detected in wild African suids (warthogs, bush pigs, and giant forest hogs), wild boars, and domestic pigs in over 60 countries (Gaudreault et al., 2020). African swine fever represents an increasing economic burden worldwide: following its emergence in China in 2018 (Ge et al., 2018), an estimated 300 million pigs have died from infection or preventive culling, with economic losses ranging in the range of 100 billion dollars. ASF is caused by African swine fever virus (ASFV), a large enveloped DNA virus belonging to the *Asfarviridae* family. Different strains of ASFV have been associated with varying levels of virulence. Highly virulent ASFV strains induce a severe disease, with clinical symptoms and pathological lesions reminiscent of hemorrhagic fevers, and mortality rates often close to 100%. In contrast, attenuated ASFV isolates only cause a moderate disease with few, and for the most part unspecific, symptoms. These different clinical outcomes may be the result of differences in the immune response to ASFV. Indeed, although infection of aortic endothelial cells has been reported (Vallée et al., 2001), the main target cells of ASFV are likely macrophages and dendritic cells (Franzoni et al., 2017, 2018a,b), which play pivotal roles in the host immune response. In these myeloid cells, virulent strains of ASFV induce high levels of inflammation, which may be deleterious to the host, while attenuated strains ASFV stimulate the secretion of type I interferon (IFN-I), which may help to control infection (Gil et al., 2008). Important genomic differences between ASFV strains have been reported: indeed, the size of ASFV genomes ranges from 170 to 194 kbp (Malogolovkin and Kolbasov, 2019). Most naturally or laboratory-attenuated ASFV strains carry large deletions and lack many so called “virulence factors” that have been reported to antagonize innate immune pathways (Dixon et al., 2019). However, basic knowledge on the cellular pathways involved in ASFV detection remains limited. In this mini-review, we summarized the current knowledge on the pattern recognition receptors (PRRs) involved in ASFV sensing, the innate immune pathways being activated following ASFV infection, and the mechanisms of viral innate immune escape.

## Activation of the cGas/Sting Pathway by African Swine Fever Virus

While ASFV infection of porcine macrophages (the main target of the virus) triggers a potent innate immune response, the PRRs involved in ASFV detection remain only partially characterized. The cGAS/STING signaling pathway has emerged as a key mediator of immunity to infection, cellular stress, and tissue damage. It has a broad capacity to sense and regulate the cellular response toward microbial and host-derived DNAs, which serve as ubiquitous danger-associated molecules (Decout et al., 2021). Recent studies indicate that the cGAS/STING pathway is stimulated upon ASFV infection (Wang et al., 2018; García-Belmonte et al., 2019), as described for many other DNA viruses (Cai et al., 2014). Interestingly, comparative structural and functional analyses of porcine, murine, and human cGAS have confirmed that these orthologs share key features (Kato et al., 2013), which may allow to extrapolate some knowledge on human cGAS to the innate sensing of other less-studied viruses such as ASFV. In addition, several ASFV virulence factors inhibit its activation: both DP96R and MGF-360-11L inhibit cGAS activation and TBK1 phosphorylation (Wang et al., 2018; Yang et al., 2021c), while MGF-505-7R and MGF-505-11R degrade STING (Li et al., 2021a; Yang et al., 2021c). Other viral proteins not previously labeled as virulence factors also have the ability to impair cGAS activation: for instance, pS273R, a viral protease, prevents the interaction between STING and IKK-ε (Luo et al., 2021). Many of these studies, however, were conducted in human cell lines and relied on overexpression approaches. Studies using specific chemical inhibitors of cGAS and/or knock-down or knock-out approaches in porcine cells are necessary to formally confirm the involvement of cGAS in ASFV detection. With so many viral proteins targeting the cGAS/STING pathway (at least when expressed ectopically), it will be interesting to determine whether cGAS activation is indeed inhibited during the course of ASFV infection and to compare the ability of virulent and attenuated strains to do so.

While the mechanisms of regulation and inhibition of cGAS have been extensively studied in humans (Hertzog and Rehwinkel, 2020; Taffoni et al., 2021), less is known about these mechanisms in pigs and other livestock species. For instance, it is unknown whether cGAS binds directly to ASFV genomes in cytoplasmic viral factories; in the nucleus, where early genome replication occurs (Simões et al., 2019); or in other subcellular compartments, where cGAS can also be detected (de Oliveira Mann and Hopfner, 2021). It would also be interesting to determine whether cGAS activation by ASFV leads (as expected) to the production of the secondary messenger cGAMP and ultimately to its passive incorporation in viral particles, as described for other viruses (Bridgeman et al., 2015).

## Potential Detection of African Swine Fever Virus by Other DNA Sensors

While the available data so far indicated a predominant role for the cGAS/STING pathway, other PRRs may also be involved in ASFV sensing. In particular, the potential roles of other DNA sensors, such as TLR9, AIM2, and IFI16, have not yet been interrogated.

TLR9 is a DNA sensor expressed in the endosomal compartment of numerous immune cell types, including porcine macrophages (Qin et al., 2016). It recognizes unmethylated CpG-rich DNA of bacterial and viral origins, as was demonstrated for example for porcine circovirus (PCV) DNA (Shi et al., 2021). TLR9 activation leads to the synthesis of proinflammatory cytokines such as IL-12, IL-6, and TNF-α, known to be induced by ASFV (Gil et al., 2008; Wang S. et al., 2021). Its involvement in ASFV detection has, however, never been directly assessed.

The family of AIM2-like receptors (ALRs) consists of four proteins in humans: MNDA, PYHIN1, IFI16, and AIM2. While the pig genome does include MNDA, PYHIN1, and IFI16, AIM2 seems to be missing (Dawson et al., 2017), suggesting that the porcine AIM2 inflammasome may not be functional (Brunette et al., 2012). However, comparison of porcine and murine AIM2 inflammasomes indicated that they are activated by the same triggers, which differ significantly from the other major inflammasome, the NLRP3-inflammasome (Kim et al., 2014; Ahn et al., 2018). This activation leads to a pathway of lytic cell death called pyroptosis, as a result of caspase-1 activation, maturation, and release of IL-1 and IL-18 and gasdermin D cleavage. ASFV inhibits this process through its viral protease pS273R, which has been reported to cleave gasdermin D in a non-canonical way that does not lead to pyroptosis (Zhao et al., 2022). ASFV also encodes many other inhibitors of apoptotic cell death, including the Bcl-2 like protein A179L; the C-type lectin AP153R; and the inhibitor of apoptosis protein (IAP) A224L (Dixon et al., 2017).

In the absence of AIM2, which other sensor may activate inflammasomes in response to ASFV infection? IFI16, which recognizes many DNA viruses (including Kaposi’s sarcoma-associated herpesvirus, Herpes simplex virus, and Epstein-Barr virus) is an interesting candidate because it is also able to detect genomic lesions following DNA damage (Taffoni et al., 2021). While ASFV replicates mostly in cytoplasmic viral factories (Netherton and Wileman, 2013), ASFV proteins p10 and p37 can accumulate in the nucleus, forming readily detectable *foci* (Eulálio et al., 2007; Nunes-Correia et al., 2008). Early ASFV genome replication events in the nucleus have been reported to induce the DNA damage response (DDR) pathway (Simões et al., 2013) and to alter the nuclear architecture and to induce changes in the epigenetic landscape (Ballester et al., 2011; Simões et al., 2015a,b, 2019). It is therefore an intruding thought that IFI16 may recognize ASFV, either directly (by binding to viral DNA) or indirectly (by recognizing ASFV-induced DNA damage). Similar to observations made in human macrophages (Jønsson et al., 2017, 16), porcine IFI16 has recently been described to regulate cGAS signaling (Zheng et al., 2020). Further studies are required to establish whether IFI16 (or other ALRs such as MNDA or PYHIN1) is involved in ASFV sensing, and whether this impacts ASFV cGAS/STING detection. While many studies in human cells suggest that cGAS is engaged in cross-talks with various sensing pathways, including some targeting RNA viruses (Anwar et al., 2021), this phenomenon remains poorly studied in pigs. If confirmed, it would then be interesting to study the effect of ASFV-encoded cGAS inhibitors on these other sensing pathways.

## Recognition of African Swine Fever Virus by Other Sensors

There is preliminary evidence that ASFV may also be detected by other PRRs beyond DNA sensors. For instance, the viral protein CD2v, an attachment factor required for ASFV infection in the *Ornithodoros* soft tick vector (Rowlands et al., 2009) stimulates IFN production (Chaulagain et al., 2021) by an unknown mechanism. AT-rich regions in ASFV DNA may also be recognized by RNA Pol III, which in turn activates RIG-I (Ran et al., 2022), although the ASFV virulence factor I267L inhibits this process by impairing Riplet-mediated activation of RIG-I (Ran et al., 2022). Several studies also suggest a role for TLR3 in ASFV detection. Indeed, its expression (together with other TLRs) is upregulated following ASFV infection (Yang et al., 2021a), and ASFV encodes two inhibitors of TLR3, A276R, and I329L. A276R inhibits IFN production following stimulation of TLR3 by poly(I:C) (Correia et al., 2013) through an unknown mechanism. I329L is a functional homolog of TLR3 and blocks IFN signaling at the level of TRIF (de Oliveira et al., 2011; Correia et al., 2013). How would TLR3, a PRR detecting dsRNAs in endosomes, be involved in the detection of ASFV, a DNA virus mostly replicating in cytoplasmic viral factories (Netherton and Wileman, 2013)? ASFV enters cells through clathrin-mediated endocytosis (Galindo et al., 2015; Hernáez et al., 2016), and ASFV infection leads to the redistribution endosomal membranes to the site of viral replication (Cuesta-Geijo et al., 2022), so it is conceivable that TLR3 ends up in proximity of ASFV RNA transcripts. To our knowledge, the production of dsRNAs was not yet observed in the context of ASFV infection but was reported for other DNA viruses (Weber et al., 2006). Future studies are therefore required to formally demonstrate the involvement of RNA sensors in ASFV sensing and to characterize at which stage of the ASFV replication cycle this detection takes place. Interestingly, multiple single nucleotide polymorphisms (SNPs) in TLRs have been observed in wild boars (Bergman et al., 2010) and in some specific breeds of domestic pigs (Palermo et al., 2009; Chen et al., 2021), and hence it would therefore be interesting to assess their effect on ASFV innate immune sensing.

## Nuclear Factor κB Activation and Stimulation of Inflammation by African Swine Fever Virus

Modulation of the inflammatory response has been suggested to be (at least in part) responsible for the observed differences in clinical outcomes following ASFV infection (Zhu et al., 2019). Indeed, *in vivo* studies showed that pigs infected with virulent ASFV exhibited a delayed onset of the inflammatory response, allowing the virus to replicate and to escape immune defenses, to later trigger a fatal cytokine storm (Wang S. et al., 2021). In this study, we discussed how ASFV modulates activation of the NF-κB signaling pathway.

The NF-κB transcription factor family is formed by five members, RelA (p65), RelB, c-Rel, p50/p105 (NF-κB1), and p52/p100 (NF-κB2) and plays a role as a master regulator of cell death and innate immune responses. It integrates the signals triggered by different PRRs to mount a suitable cellular response (Dorrington and Fraser, 2019). This makes this pathway a perfect target for viruses which have evolved diverse strategies to dampen or modulate the cellular innate immune response it in order to promote viral replication (Hayden and Ghosh, 2008; Schmitz et al., 2014). This is also the case for ASFV. However, NF-κB modulation by ASFV is a double-edged sword: on the one hand, the virus exploits the antiapoptotic functions of NF-κB to promote early viral replication; on the other hand, ASFV employs mechanisms to evade NF-κB-mediated antiviral cytokine responses. Primary alveolar macrophages (the most suitable cellular infection model) infected with highly virulent ASFV display a strong upregulation of genes belonging to this pathway (Cackett et al., 2022) and blocking its activation with the inhibitor BAY11-7082 limits viral replication *in vitro* (Gao et al., 2022). Upon PRR stimulation, signal transduction will activate the IKK complex (formed by IKK-α, IKK-β, and IKK-γ), which, in turn, will phosphorylate IκB proteins, leading to their degradation and the release of free NF-κB dimers that will translocate to the nucleus. There, NF-κB will trigger the expression of proinflammatory cytokines and negative feedback loops to ultimately shut down the inflammatory response (Ashall et al., 2009). Thus, rather than acting as a simple on/off switch for inflammatory responses, NF-κB activity is an intricate, tightly regulated and dynamic process. This inflammatory response needs to be strong enough to eliminate the infection, but at the same time, it must not cause too much damage to the host (Gottschalk et al., 2016). Single cell studies show that differences in NF-κB signaling dynamics will lead to differences in the subset of genes expressed by the cell (Tay et al., 2010; Kellogg et al., 2017; Lane et al., 2017). NF-κB activation dynamics seem to be cell- and stimulus-dependent for instance, macrophages show a gradual activation of NF-κB with a long-lasting nuclear translocation, while non-inflammatory cells seem to activate it in an “all or nothing” manner showing NF-κB oscillations (Dorrington and Fraser, 2019; López-Gálvez et al., 2021).

African swine fever virus disrupts NF-κB signaling at different levels. The ubiquitin-conjugating enzyme (UBCv1) and F317L are early viral proteins blocking NF-κB activation upstream or at the level of the IKK complexes (Barrado-Gil et al., 2021; Yang et al., 2021b). F317L interacts with IKK-β to block IκBα phosphorylation, and its overexpression promotes viral replication (Yang et al., 2021b). ASFV also blocks NF-κB signaling through the A238L protein, an IκBα homolog binding directly to NF-κB heterodimers and inhibiting their nuclear translocation (Powell et al., 1996; Silk et al., 2007). Furthermore, A238L has been suggested to interact with the CBP/P300 transcription coactivator proteins (Granja et al., 2006), which are involved in the regulation of NF-κB-dependent transcription (Mukherjee et al., 2013) to inhibit the synthesis of TNF-α. Interestingly, many ASFV genes do not only block NF-κB activation but also fulfill other functions. For instance, A224L is an inhibitor of apoptosis (IAP)-like protein, which also activates NF-κB signaling (Rodríguez et al., 2002). In addition, the K205R protein triggers endoplasmic reticulum stress and subsequently increases NF-κB activation (Wang Q. et al., 2022). Another multifunctional ASFV viral protein is MGF-505-7R (Huang et al., 2022), which, in addition to its ability to target the IFN pathway, suppresses TLR8-mediated phosphorylation of p65 phosphorylation (Liu X. et al., 2021) and inhibits IL-1 production by interfering with the formation of the NLRP3 inflammasome (Li et al., 2021c).

Altogether, ASFV infection results in a complex interactome of viral proteins that can activate or inhibit NF-κB. Further studies are needed to better understand how this mosaic of interactions modulate NF-κB and determine cell fate. A key question would be to understand the mechanisms through which virulent ASFV strains overcome the cellular regulatory “checkpoints” and shift the immune response from a low-degree inflammation to a sudden (and fatal) cytokine storm. For instance, assessing the kinetics of expression of virulence genes across different ASFV strains would allow to build a model explaining how virulent strains manipulate the early inflammatory response and trigger excessive cytokine production leading to endothelial dysfunction, coagulopathy and ultimately fatal hemorrhages. Future studies may also assess whether differences in NF-κB pathway activation can be responsible for the absence of symptoms in some ASFV-infected animals such as wild African suids. Although one report has demonstrated that warthog-specific signatures in RelA/p65 (Palgrave et al., 2011) do not confer ASFV resistance to domestic pigs (McCleary et al., 2020), it is still conceivable that other components in the NF-κB pathway may differ between warthogs (or other wild African suids) and pigs, either at the genetic level or in their activation/regulation dynamics.

## Viral Antagonism of Interferon and Nuclear Factor κB Pathways and Virulence: a Complex Picture

As discussed earlier, several ASFV viral genes seem to be able to prevent the induction of the cGAS/STING pathway. In addition, many other ASFV genes have a more downstream effect on IFN signaling. Early genomic studies comparing ASFV genomes from virulent and attenuated ASFV have identified genes of the multi gene family (MGF) 360 and 505 as being deleted in attenuated ASFV (Neilan et al., 2002; Chapman et al., 2008; Portugal et al., 2015). Consistent with this, attenuated strains lacking genes from this family appear to be unable to shut down the IFN response (Portugal et al., 2018) and to be more sensitive to its antiviral effects (Golding et al., 2016). Deletion of MGF 360/505 genes from virulent ASFV strains led to attenuation *in vivo* (O’Donnell et al., 2015) confirming the relevance of this gene family. This prompted intense research efforts to uncover which virulence factors are responsible for the modulation of the innate immune response to ASFV, although the relative contributions of each individual gene remain elusive. In addition, the relationship between ASFV pathogenicity and viral replication is complex, as many attenuated ASFV strains are still able to sustain high levels of replication, at least *in vitro* (Lacasta et al., 2015; Reis et al., 2016; Franzoni et al., 2018b,^2020^). Since several good reviews have been published recently covering these topics (Dixon et al., 2019; Gladue and Borca, 2022; Wang Z. et al., 2022), we did not aim to make an exhaustive list of the ASFV genes targeting innate immune pathways but rather highlight recent discoveries and illustrate conserved antagonism strategies.

Recent study has suggested that MGF-505-1R and MGF-360-12L both inhibit the IFN response, albeit with different mechanisms, as only the latter impacted ASFV replication in macrophages (Rathakrishnan et al., 2022). Strikingly, some key proteins of the IFN pathway are the target of multiple ASFV inhibitors. For instance, TBK1 is inhibited (or degraded) by E120R (Liu H. et al., 2021), DP96R (Wang et al., 2018), MGF-360-11L (Yang et al., 2022), and A137R (Sun et al., 2022). Similarly, JAK/STAT are targeted by MGF-360-9L (Zhang et al., 2022) and MGF-505-7R (Li et al., 2021b). Some ASFV proteins, like I226R (Hong et al., 2022), I329L (de Oliveira et al., 2011), and MGF-360-12L (Zhuo et al., 2021) are able to target both the IFN and NF-κB pathways at the same time. Interestingly, multiple ASFV proteins, such as A137R (Sun et al., 2022), K205R (Wang Q. et al., 2022), and MGF-505-7R (Li et al., 2021a), induce the autophagy pathway, which may represent a strategy shared by multiple virulence genes to modulate the innate immune response to ASFV.

As summarized in Table 1, many ASFV viral genes modulate IFN and NF-κB signaling. Such a level of functional redundancy likely indicates that ASFV is under strong selective pressure to evade the antiviral effects of IFN and NF-κB. To date, however, only a very limited subset of IFN-stimulated genes (ISGs) have been studied in the context of ASFV. So far, only MxA (Netherton et al., 2009) and IFITM2/3 (Muñoz-Moreno et al., 2016) have been described to inhibit the replication of Ba71V, the Vero-adapted strain of ASFV. Whether ASFV evolved mechanisms of antagonism of antiviral ISGs (for instance through virulence factors) has not been studied either.

**TABLE 1:** Examples of African swine fever virus (ASFV) viral genes modulating interferon (IFN) and nuclear factor kappa B (NF-κB) activation.

Viral gene	Effect on virulence	Mechanism of action	References
S2736	Unclear	Inhibition of the IKKε/STING interaction through IKKε binding. Involved in pyroptosis modulation	Luo et al., 2021; Zhao et al., 2022
MGF-505-7R	Deletion leads to attenuation in the HLJ/18 strain	Upregulation of ULK1 and degradation of STING through autophagy. Interaction with IKKα inhibiting NF-κB activation	Li et al., 2021a,b
I267L	Deletion leads to attenuation in the CN/GS/201 strain	Inhibition of Riplet-mediated K63-polyubiquitination and activation of Rig-I	Ran et al., 2022
MGF-360-9L	Deletion leads to attenuation in the CN/GS/201 strain	Degradation of STAT1 and STAT2	Zhang et al., 2022
MGF-360-11L	Unclear	Degradation of TBK1 and IRF7	Yang et al., 2022
MGF-360-14L	Unclear	Degradation of IRF3 through TRIM21-mediated K63-ubiquitination	Wang Y. et al., 2021
MGF-505-11R	Unclear	Degradation of STING	Yang et al., 2021c
MGF-360-12L	Deletion leads to attenuation in Georgia strain	Blocks interaction between importins and NF-κB	Zhuo et al., 2021; Rathakrishnan et al., 2022
K205R	Unclear	Stimulation of p65 nuclear translocation. Involved in ER stress and autophagy	Wang Q. et al., 2022
E120R	Unclear	Interaction with IRF3 blocking its activation	Liu H. et al., 2021
DP96R	Deletion leads to attenuation in some (but not all) strains	Inhibition of TBK1 phosphorylation	Wang et al., 2018; Ramirez-Medina et al., 2019
A238L	Deletion in the E70 isolate has no effect	IκBα homolog binding inhibiting NF-κB translocation. Interaction with CBP	Powell et al., 1996; Granja et al., 2006; Silk et al., 2007; Salguero et al., 2008

*For the indicated ASFV genes is indicated the effect on virulence (e.g., when deleted from virulent strains), the proposed mechanism of action and the references.*

Attenuated and virulent strains of ASFV seem to differ in the innate immune response they induce, with different profiles of cytokines being produced after infection (Gil et al., 2008). However, the role of individual ASFV virulence genes remains unclear. Indeed, while deletion of some viral genes, such as I267L (Ran et al., 2022), MGF-505-5R (Li et al., 2021a, c; Huang et al., 2022), MGF-360-12L, and MGF-505-1R (Rathakrishnan et al., 2022) lead to ASFV attenuation *in vivo*, others, such as I329L (Reis et al., 2020) or A238L (Salguero et al., 2008), seem to have no effect. Moreover, these effects can be strain-dependent: for instance, deletion of DP96R lead to attenuation in the virulent strain E70 but not in the virulent Georgia strain that emerged in Europe in 2007 (Ramirez-Medina et al., 2019). In some cases, deletion of viral genes known to modulate the immune response had deleterious effects on the efficacy of live attenuated vaccines candidates (Reis et al., 2020; Lopez et al., 2021; Rathakrishnan et al., 2022). These studies highlight the complexity of the mechanisms underlying ASFV virulence and suggest that multiple viral proteins act in concert to shape the innate immune response to ASFV.

## Conclusion

**FIGURE 1:**
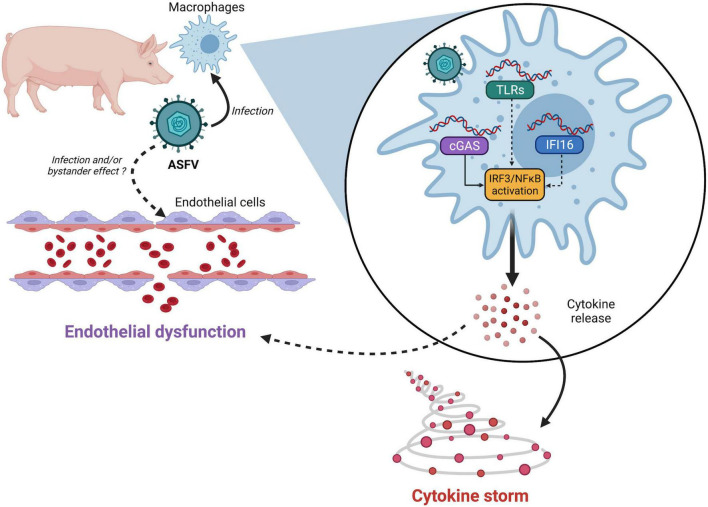
Links between African swine fever virus (ASFV) sensing and pathogenesis. ASFV nucleic acids—either incoming DNA or newly synthesized viral DNA/RNA—may be detected by several pattern recognition receptors (PRRs). While cGAS has been validated by multiple teams, the involvement of other PRRs like IFI16 or TLRs cannot be ruled out. ASFV detection then triggers IRF3 and NF-κB activation resulting in uncontrolled secretion of proinflammatory cytokines. Whether endothelial dysfunction is mediated by apoptosis of infected cells or through bystander effects remains to be determined.

The mechanisms of innate immune detection of ASFV are just starting to be described. While most studies focused on the cGAS/STING pathway so far, ASFV may also be sensed by other DNA sensors such as TLR9 or IFI16 or by RNA sensors like TLR3 or RIG-I. Many ASFV genes (including virulence factors but also conserved genes) inhibit the activation of the IRF3 and NF-κB signaling pathways at different levels. Such functional redundancy strongly suggests that ASFV is under strong pressure to antagonize these pathways, although the roles of individual genes (and their mechanisms) remain to be fully characterized. It will be of particular interest to determine which viral genes shape the innate immune response to ASFV, and how this process leads to endothelial dysfunction and cytokine storm, the main hallmarks of virulent ASFV infection (Figure 1). While direct infection of endothelial cells has been reported to trigger apoptosis (Vallée et al., 2001), endothelial dysfunction may also be an indirect consequence of excessive proinflammatory cytokine production (Tatoyan et al., 2019). Further studies may also shed light on the mechanisms responsible for the lack of virulence of some naturally attenuated ASFV strains in domestic pigs, as well as for ASFV persistence in wild African suids, which do not develop symptoms upon infection (Oura et al., 1998). Ultimately, research efforts exploring the complex mechanisms of ASFV pathogenicity may guide the design of safe and robust live attenuated vaccines for this deadly pathogen.

## Author Contributions

AA, ST, RG, IC, and FR wrote and proofread the manuscript and approved the submitted version.

## Conflict of Interest

The authors declare that the research was conducted in the absence of any commercial or financial relationships that could be construed as a potential conflict of interest.

## Publisher’s Note

All claims expressed in this article are solely those of the authors and do not necessarily represent those of their affiliated organizations, or those of the publisher, the editors and the reviewers. Any product that may be evaluated in this article, or claim that may be made by its manufacturer, is not guaranteed or endorsed by the publisher.
